# Complex Fiber Micro-Knots

**DOI:** 10.3390/s18041273

**Published:** 2018-04-20

**Authors:** Shir Shahal, Hamootal Duadi, Yoav Linzon, Moti Fridman

**Affiliations:** 1 Faculty of Engineering and the Institute of Nanotechnology and Advanced Materials, Bar-Ilan University, Ramat Gan 5290002, Israel; shirshahal@gmail.com (S.S.); hamootal@gmail.com (H.D.); 2 School of Mechanical Engineering, Faculty of Engineering, Tel Aviv University, Tel Aviv 69978, Israel; yoav.linzon@gmail.com

**Keywords:** fiber micro-knot, long-period fiber grating, high-order fiber modes

## Abstract

Fiber micro-knots are a promising and a cheap solution for advanced fiber-based sensors. We investigated complex fiber micro-knots in theory and experiment. We compared the measured spectral response and present an analytical study of simple micro-knots with double twists, twin micro-knots, figure-eight micro-knots, and tangled micro-knots. This research brings the simple fabrication process and robustness of fiber micro-knots into the world of complex resonators which may lead to novel optical devices based on fiber micro-knots.

## 1. Introduction

Fiber micro-knots are fabricated by tying a knot in a micro-fiber and carefully pulling it until it reaches a micrometer scale [1]. Fiber micro-knots were suggested more than a decade ago [2] and have drawn much attention due to their simplicity, low price, and compact size [1]. Fiber micro-knots are sensitive to external conditions such as temperature, motion, or any change in refraction index [3], so they have been implemented in a variety of sensors [4,5,6,7,8,9,10,11,12,13].

However, while complex resonators have been introduced in silicon photonics [14,15,16,17], only simple knots have been introduced in fiber micro-knots [18,19,20,21,22]. Most micro-knot devices are based on a single knot [18,23], and although some have demonstrated the coupling of two knots [24] or a single knot combined with a Mach–Zehnder interferometer [20], they have required complex fabrication techniques. Other techniques for multiple resonators are to coil the fiber around a ring [25] or to utilize the whispering gallery modes in fibers [26] but in both techniques the diameter is not tunable and the complexity of the fiber device is limited. Therefore, we fabricated complex fiber micro-knots that combine several coupled micro-knot devices and measured their spectral response. We developed a technique to analyze the different devices based on splitting the full scheme into small segments and show good agreement between the measured and calculated results. This research can lead to the implementation of fiber micro-knots in photonic devices, even when complex resonators such as high-end sensors are needed. Such micro-knot-based devices will be easier to fabricate, with improved robustness, stability, and durability, and can reduce the fabrication cost.

## 2. A Simple Micro-Knot

For completeness, we first analyze a simple fiber micro-knot [27]. We start by tapering a fiber down to a 6 μm width over a length of 30–50 mm [28,29]. When a fiber is tapered below 20 μm, the light is no longer guided by the core but by the cladding, which leads to an evanescence field outside the fiber. Thus, when placing two tapered fibers next to each other, the light couples from one fiber to the other, where the coupling strength, κ, depends exponentially on the fibers diameter [30].

When tying a knot in a tapered fiber, the light couples between the touching fibers and resonates around the micro-knot, which results in a ring resonator [1]. The transmission, $E_{out}$, of this resonator as a function of the propagating vector, *k*, is calculated by [31]

(1) $$E_{out}=\frac{{\left(1-\kappa \right)}^{2}}{1-\kappa e^{ikl}}$$

where *l* is the length of the micro-knot cavity.

**Figure 1:**
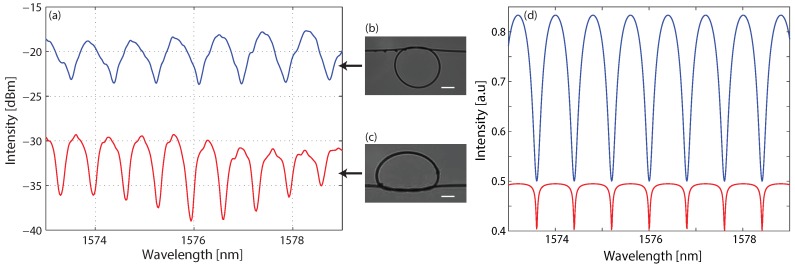
Transmission spectra of two simple micro-knots. (**a**) Experimental results where the upper spectrum presents the transmission spectrum of a simple micro-knot with a single twist, as shown in inset (**b**); the lower spectrum presents the transmission spectrum of a double twist micro-knot, as shown in inset (**c**). The scale bars are 200 μm. (**d**) Numerical simulations of two micro-knots, one with lower losses and lower coupling strength denoted by the blue curve and one with higher losses with higher coupling strength denoted by the red curve.

We fabricated a simple micro-knot as presented in Figure 1b and measured the transmission spectrum, which is presented in the upper spectrum in Figure 1a. The measured free-spectra range (FSR) of 0.86 nm, and the micro-knot diameter of 600 μm, measured by the microscope picture, agree according to Equation (1). We also fabricated a simple micro-knot with a double twist, as shown in Figure 1c. The double twist increases the coupling strength as evident by the deeper and sharper resonances and decreased free-spectral range presented in the lower spectrum in Figure 1a. However, the double twist distorts the micro-knot shape, leading to higher losses and lower overall transmission. Additionally, due to the shape distortion, the minimal diameter of the double twist micro-knot was 800 μm. We numerically simulated the two micro-knots according to Equation (1) and present the results in Figure 1d. We simulated two micro-knots, one with lower coupling strength and lower losses denoted by the blue curve and one with higher coupling strength and higher losses denoted by the red curve.

## 3. Complex Micro-Knots

We developed and fabricated micro-knots with higher complexity: twin micro-knots, a figure-eight micro-knot, and a tangled micro-knot.

**Figure 2:**
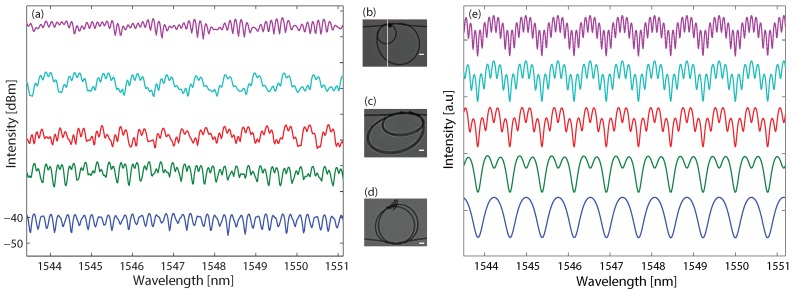
Transmission spectra of twin micro-knots with different size ratios. (**a**) Experimental measurements; (**e**) numerical simulations. The curves, from top to bottom, represent the transmission spectra of twin micro-knots with size ratios of 1:7, 1:5, 1:4, 1:3, and close to 1:1, respectively. Insets shows typical twin micro-knots pictures with size relations of 1:3 (**b**), 1:2 (**c**), and close to 1:1 (**d**). The scale bars are 200 μm.

The twin micro-knots were fabricated by combining two simple micro-knots of different sizes, as presented in the insets of Figure 2. The two micro-knots are coupled together, so the output field $E_{out}$ as a function of the wave vector *k* is as follows [32]:

(2) $$E_{out}=\frac{{\left(1-\kappa \right)}^{4}}{\left(1-\kappa e^{ikl_{1}}\right)\left(1-\kappa e^{ikl_{2}}\right)}$$

where κ is the coupling strength between adjacent fibers, $l_{1}$ is the length of one of the micro-knots, and $l_{2}$ is the length of the other micro-knot. The resulting spectral response is obtained by multiplying two transmission spectra of a single micro-knot, resulting in beating in the frequency domain [33,34,35,36,37,38,39]. Therefore, each micro-knot generates a spectral response similar to the one presented in Equation (1), where the size of the micro-knot determines the free-spectral range and the coupling strength determines the resonances shape.

We measured the transmission spectrum as a function of the size ratio and present the results in Figure 2a. The curves, from top to bottom, represent the transmission spectra of twin micro-knots with size ratios of 1:7, 1:5, 1:4, 1:3, and close to 1:1, respectively. The spectra curves are shifted compared to the lowest curve to prevent overlapping. At the insets, we present typical pictures of twin micro-knots with size ratios of 1:3 (b), 1:2 (c) and close to 1:1 (d). Twin micro-knots with higher ratios did not fit into the microscope field of view. We numerically simulated the transmission spectrum according to Equation (2) with the same size ratios. The results are presented in Figure 2e and are similar to the experimental results.

**Figure 3:**
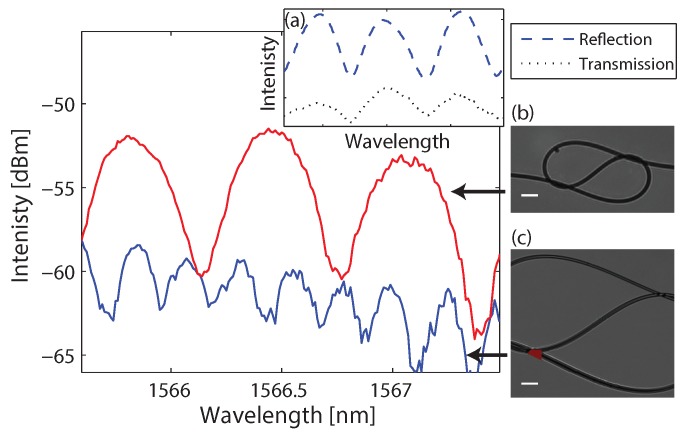
Reflection spectra of figure-eight micro-knots with different sizes. The micro-knots pictures are presented in insets (**b**,**c**). Inset (**a**) shows the measured transmission spectrum and reflection spectrum of the same micro-knot showing similar spectral responses. The scale bars are 200 μm.

In many cases, it is possible to access only one end of the fiber. Therefore, we developed and fabricated a figure-eight micro-knot in which the reflection spectral response is identical to the transmission spectral response. A typical picture of a figure-eight micro-knot is shown in Figure 3b. Light which couples into a figure-eight micro-knot resonates in a counterclockwise rout until it goes through the middle fiber. Then, the light resonates in a clockwise rout until it couples out to either directions. Therefore, the transmission and the reflection spectra are identical up to a phase. The light cannot couple back to a counterclockwise resonance. According to the analysis of the beam rout in the fiber, we evaluated the spectral response of this micro-knot and obtained that the output field $E_{out}$ as a function of the wave vector *k* up to a phase is

(3) $$E_{out}=\frac{{\left(1-\kappa \right)}^{4}}{{\left(1-\kappa^{2}e^{ikl}\right)}^{2}}$$

where κ is the coupling strength between adjacent fibers, and *l* is the length of the ring cavity, namely the length of the micro-knot without the middle fiber.

First, we verified that the transmission and the reflection have the same spectral response, and present the measured spectra in Figure 3a. Next, we measured the reflection spectrum as a function of the micro-knot size and observed the same behavior of increased FSR when shrinking the micro-knot as predicted. We compared the FSR to the measured micro-knot size and found that they agree with Equation (3). This figure-eight micro-knot can also be beneficial for fiber laser gyroscope and motion sensors since the light resonates both clockwise and counterclockwise.

**Figure 4:**
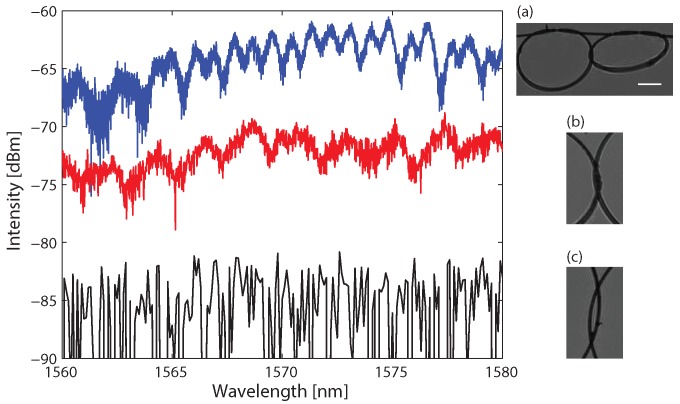
Reflection and transmission spectra of tangled micro-knots. Upper curve (blue)—transmission spectrum, middle curve (red)—reflection spectrum when the two micro-knots are touching, lower curve (black)—reflection spectrum when the two micro-knots are not touching showing no reflected light from the micro-knot. Inset (**a**) shows a typical picture of a tangled micro-knot. Insets (**b**,**c**) show representative zoomed pictures of the touching area between the two micro-knots: (**b**) when the two micro-knots are touching; (**c**) when the two micro-knots are not touching. The scale bar is 200 μm.

Finally, we demonstrate twin micro-knots with both reflection and transmission spectral responses. We fabricated twin micro-knots where the two micro-knots are tangled together as demonstrated in Figure 4a. Analyzing this tangled micro-knot is similar to the figure-eight micro-knot. When the light couples into the micro-knot, it resonates clockwise on the left micro-knot and counterclockwise on the right micro-knot. After the light travels once in the short fiber between the micro-knots it resonates in the opposite directions and can exit the tangled micro-knot in either directions. Similar to the figure-eight micro-knot, the light cannot travel again in the short fiber. This enables us to evaluate the output field $E_{out}$ as a function of the wave vector *k* up to a phase as

(4) $$E_{out}=\frac{{\left(1-\kappa \right)}^{4}\kappa^{4}}{{\left(1-\kappa \left(1-\kappa \right)e^{ikl_{left}}\right)}^{2}{\left(1-\kappa \left(1-\kappa \right)e^{ikl_{right}}\right)}^{2}}$$

where κ is the coupling strength between the fibers, $l_{left}$ is the cavity length of the left micro-knot, and $l_{right}$ is the cavity length of the right micro-knot. We measured the transmission spectral response of the tangled micro-knot and observed the double periodicity, as presented in the upper curve (blue) in Figure 4.

This tangled micro-knot is harder to fabricate and resulted in higher losses. Specifically, it was challenging to adjust the two micro-knots to touch each other. When the two micro-knots touched as shown in Figure 4b, we measured reflection spectral response, presented in the middle curve (red) in Figure 4. However, when the two micro-knots did not touch, as shown in Figure 4c, there was no reflection from the tangled micro-knot. This is verified by the fact that the measured spectral response results in the noise level of the optical spectrum analyzer and is presented in the lower curve (black) in Figure 4. The numerical simulations of the figure-eight micro-knot and the tangled micro-knot are identical to the square of the numerical simulations of the simple micro-knot and the twin micro-knots, respectively.

## 4. Discussion and Conclusions

We summarized the results in Table 1 comparing the five different micro-knots. We show the free-spectral range, Q-factor, spectral amplitude, and the losses. We note that, in our demonstration, the losses are too high for practical use, especially with the micro-knot with higher complexity. Nevertheless, we are working on improved fabrication techniques to reduce the losses and such micro-knots can still be useful for situations where losses are less important such as chemical sensors where strong lasers can be used.

A simple micro-knot can be implemented when a simple resonator is needed and the double twist increases the coupling without resorting to thinner fibers, which are more fragile. When a spectral response with higher complexity is required, as is the case in chemical sensors, few micro-knots, such as our double micro-knot, can be implemented. When only one side of the fiber is available, a figure-eight micro-knot or a tangled micro-knot that has higher spectral complexity can be utilized. A variety of different micro-knots devices can be built from these building blocks and can be implemented in optical data processing, quantum optical manipulation devices, specific sensors, and photonic processing devices.

To conclude, we fabricated, analyzed, and investigated four types of complex fiber micro-knots. We demonstrated simple micro-knots with increased coupling strength by twisting the fiber twice instead of once. We presented twin micro-knots and showed the beating in the frequency domain, which can be implemented in complex and tunable filters. In addition, we developed figure-eight micro-knots and tangled micro-knots with similar reflection and transmission spectral response for situations, where only one end of the fiber is accessible. We hope that these results will enable the integration of complex fiber micro-knots in detectors and sensing devices with optical data processing systems, which cannot be done with simple micro-knots resonators.

## Figures and Tables

**Table 1:** The properties of different types of micro-knots.

Type	Picture	FSR	Q-Factor	Amplitude	Losses
simple knot	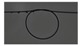	0.87 nm	4600	5.5 dB	10 dB
double twist	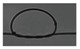	0.66 nm	11,000	9 dB	20 dB
double knot	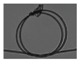	0.2 nm	30,000	8 dB	30 dB
figure-eight	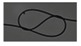	0.63 nm	9000	8 dB	45 dB
tangled	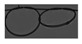	0.9 nm	1500	3 dB	55 dB
